# Anti-*Borrelia* IgG Seropositivity Among Hemodialysis Patients with Chronic Kidney Disease of Unknown Etiology: A Preliminary Case–Control Study from Northern Türkiye

**DOI:** 10.3390/pathogens15060655

**Published:** 2026-06-22

**Authors:** Eşe Aslan Başbulut, Demet Yavuz, Tülin Demir

**Affiliations:** 1 Department of Medical Microbiology, Samsun Training and Research Hospital, Health Science University, Samsun 55090, Türkiye; eseasla@yahoo.com; 2 Department of Nephrology, Faculty of Medicine, Samsun University, Samsun 55090, Türkiye; demetdolu@hotmail.com; 3 Department of Clinical Microbiology, Faculty of Medicine, Ahi Evran University, Kirsehir 40100, Türkiye

**Keywords:** *Borrelia burgdorferi*, chronic kidney disease, chronic renal failure, Lyme borreliosis, seroprevalence, Western blot

## Abstract

Background: Lyme borreliosis is a multisystemic tick-borne infection caused by *Borrelia burgdorferi* sensu lato complex. Renal involvement is well documented in dogs with Lyme nephritis, whereas human renal manifestations have been reported only rarely, mainly as isolated case reports of glomerular disease. Because a proportion of chronic kidney disease (CKD) cases remain unexplained, we aimed to determine the possible contribution of previous exposure to idiopathic CKD. Methods: The study included 45 patients and 45 age and sex matched controls. Serum samples were tested for anti-*Borrelia* IgG using enzyme-linked immunosorbent assay (ELISA). Positive or borderline ELISA results were further evaluated by Western blot (WB) analysis. Results: Overall, anti-*Borrelia* IgG seropositivity was 5.55% with WB. In the CKD group, ELISA positivity was 11.1% and WB-confirmed positivity was 8.9%. In the control group, ELISA positivity was 4.4% and WB-confirmed positivity was 2.2%. (*p* > 0.05). All confirmed patients were living in rural areas, engaged in farming and/or animal husbandry, and reported repeated tick exposure. Conclusions: IgG seropositivity was numerically higher among patients with CKD of unknown etiology than among healthy controls; however, this difference was not statistically significant. Larger, risk-factor-adjusted studies are needed to clarify whether *Borrelia* exposure may be associated with unexplained CKD.

## 1. Introduction

Lyme borreliosis is a tick-borne infection caused by spirochetes belonging to the *Borrelia burgdorferi* sensu lato complex. It is considered the most common vector-borne infection in the Northern Hemisphere and represents an important public health concern in endemic and emerging regions. Environmental and climatic changes may influence tick distribution, tick abundance, and the seasonal dynamics of tick-borne infections, thereby contributing to increased recognition of Lyme borreliosis in recent years [1,2,3,4].

The clinical spectrum of Lyme borreliosis is broad. Early disease often presents with erythema migrans, whereas untreated or delayed cases may involve the musculoskeletal, nervous, cardiovascular, and dermatological systems. Although renal involvement is not typically considered a prevalent manifestation of Lyme borreliosis in humans, there have been isolated case reports and small case series describing various glomerular diseases. Nonetheless, these occurrences remain uncommon and inadequately characterized. Consequently, the existing evidence does not support establishing a causal relationship between *Borrelia* infection and glomerular disease [5,6,7,8,9,10,11,12,13,14].

In contrast to the limited literature on human cases, Lyme nephritis is a recognized complication in dogs. Canine Lyme nephritis is typically characterized by glomerular injury and protein-losing nephropathy and has been associated with *Borrelia burgdorferi* exposure in endemic areas [15,16,17]. While findings from canine studies cannot be directly applied to humans and their relevance to humans is limited, they support further research to investigate whether *Borrelia* exposure is associated with renal involvement in selected human populations.

Chronic kidney disease can result from several well-established etiologies, including diabetes mellitus, hypertension, glomerulonephritis, obstructive nephropathy, vasculitis, amyloidosis, malignancy, and chronic infections. Nevertheless, a considerable proportion of patients with CKD remain without a clearly identifiable cause despite clinical evaluation [18,19]. Although current evidence is limited to case reports and small case series, infectious processes that can cause immunecomplex-mediated renal damage, such as those identified in *Borrelia*-associated glomerular disease, may be considered among the potential contributing factors to renal dysfunction in a subgroup of patients with chronic kidney disease of unknown etiology. *Borrelia* infection has been associated with immunecomplex-mediated glomerular diseases, including membranoproliferative glomerulonephritis, IgA nephropathy, and membranous nephropathy [8,9,11].

The objective of this study was to evaluate the prevalence of anti-*Borrelia* IgG seropositivity in patients with CKD of unknown etiology and to compare it with healthy controls. We also aimed to describe exposure-related characteristics among seropositive CKD patients. Given the cross-sectional design of the study, the findings were interpreted as preliminary evidence of prior *Borrelia* exposure rather than as proof of causality or active Lyme-related renal disease.

## 2. Materials and Methods

### 2.1. Study Design and Participants

This single-center, observational, cross-sectional case–control study was conducted at the dialysis center of Samsun Training and Research Hospital between January and July 2022. During the study period, 161 patients were receiving hemodialysis at the center. Patients were screened for CKD etiology through detailed anamnesis, review of medical records, and evaluation of the hospital electronic patient record system and the Ministry of Health e-Pulse system.

Patients were eligible for inclusion in the CKD group if they were at least 18 years old, were receiving hemodialysis for CKD of unknown etiology, and provided written informed consent. Chronic Kidney Disease (CKD) of unknown etiology was defined as a condition in which the underlying cause of CKD cannot be determined, despite clinical evaluation, laboratory tests, imaging studies, and review of existing medical records, and cannot be classified by our nephrology specialist. Patients with known causes of CKD, including diabetes mellitus, hypertension, pyelonephritis, vasculitis, renal artery stenosis, malignancy, obstructive nephropathy, amyloidosis, or primary glomerulonephritis, were excluded. Of 52 patients identified with CKD of indeterminate etiology, seven declined participation, and 45 were enrolled.

The control group consisted of 45 healthy hospital staff members recruited from the same geographic region as the CKD patients. Controls were selected using frequency matching to achieve a similar age and sex distribution and a comparable sample size to that of the CKD group. The absence of CKD or other known chronic illnesses was determined from medical history and self-report. Specific screening for previous Lyme disease, tick exposure, serum creatinine, eGFR, or urinalysis was not performed. All participants provided written informed consent prior to enrollment. The confidentiality of participant data was maintained throughout the study.

### 2.2. Sample Collection and Storage

Five milliliters of whole blood were collected from each participant for assessment of anti-*Borrelia* IgG antibodies. Blood samples were centrifuged at 3000 rpm for 10 min. Serum samples were separated, transferred into Eppendorf tubes, and stored at −80 °C until analysis. On the day of testing, samples were thawed at room temperature and processed according to the assay procedures.

### 2.3. Anti-Borrelia IgG ELISA

Anti-*Borrelia* IgG levels were determined using a commercial enzyme-linked immunosorbent assay kit (Anti-*Borrelia* IgG ELISA, Euroimmun, Lübeck, Germany) according to the manufacturer’s instructions. The ELISA plates were coated with antigen extracts of *Borrelia burgdorferi* sensu stricto, *Borrelia afzelii*, and *Borrelia garinii*. Results were interpreted semi-quantitatively by calculating the ratio of the sample absorbance to the calibrator absorbance. Ratios < 0.8 were considered negative, ratios ≥ 0.8 and <1.1 were considered borderline, and ratios ≥ 1.1 were considered positive.

### 2.4. Borrelia IgG Western Blot Confirmation

Because ELISA alone may yield false-positive results in Lyme borreliosis serology, all samples with positive or borderline ELISA results were further evaluated using a *Borrelia* Western blot IgG assay (Euroimmun, Germany), performed according to the manufacturer’s instructions. Results were evaluated visually and digitally using the EUROLINEScan program (Euroimmun, Germany). The interpretation included reactivity against p83, p39, p41, p31, p30, OspC/p25, p21, p19, p17, and VlsE antigens, in accordance with the manufacturer’s criteria.

For the purpose of final evaluation, seropositivity for anti-*Borrelia* IgG was defined as a positive or borderline ELISA result supported by a positive Western blot. Results that were positive in both ELISA and Western blot tests were considered anti-*Borrelia* IgG seropositive.

### 2.5. Statistical Analysis

Statistical analyses were performed using SPSS version 20.0 (SPSS Inc., Chicago, IL, USA). The distribution of continuous variables was assessed using the Shapiro–Wilk and Kolmogorov–Smirnov tests. Descriptive statistics were expressed as mean ± standard deviation or median (minimum–maximum), depending on distribution. Categorical variables were summarized as frequency and percentage. Differences between categorical variables were analyzed using Pearson’s chi-square test or Fisher’s exact test, as appropriate. A *p*-value < 0.05 was considered statistically significant.

### 2.6. Ethical Approval

The study was approved by the relevant ethics committee of Saglik Bilimleri University Samsun Training and Research Hospital under protocol number SBU SEAH-GOKA/2021/16/1 on 29 December 2021. The research was conducted in accordance with the principles of the Declaration of Helsinki. Informed consent was obtained from all subjects involved in this study.

## 3. Results

During the study period, 161 patients were receiving hemodialysis at the dialysis center. Among them, 52 patients (32.3%) had CKD of indeterminate etiology. Seven of these patients declined to participate, and 45 patients were enrolled in the CKD group. The control group included 45 healthy individuals without CKD or known chronic disease. In total, 90 participants were included in the analysis (Table 1).

The mean age was 60.07 ± 11.74 years in the CKD group and 57.42 ± 7.79 years in the control group. There was no statistically significant difference between the groups in terms of age (*p* = 0.212) or sex distribution (*p* = 0.830) (Table 2).

Anti-*Borrelia* IgG ELISA testing identified seven positive participants among the 90 individuals included in the study (7.78%). In our study, all seven ELISA-positive samples were subsequently tested by Western blot. Five of these seven samples were confirmed positive by Western blot, while two were negative. No borderline ELISA and Western blot results were observed. For the final analysis, only samples confirmed by Western blot were considered seropositive. Western blot-confirmed anti-*Borrelia* IgGpositivity in five participants overall (5.55%).

In the CKD group, ELISA positivity was detected in five of 45 patients (11.1%), whereas Western blot-confirmed positivity was detected in four of 45 patients (8.9%). In the control group, ELISA positivity was detected in 2 of 45 individuals (4.4%), and Western blot-confirmed positivity was detected in 1 of 45 individuals (2.2%). In the CKD group, 4 of 45 patients tested positive by Western blot analysis, whereas only 1 of 45 patients in the control group tested positive. The CKD group had 4/45 Western blot-positive patients versus 1/45 controls. Western blot-confirmed positivity was numerically more frequent in the CKD group than in controls, but this difference did not reach statistical significance (*p* = 0.361). For Western blot-confirmed seropositivity, the odds ratio was 4.29 (95% CI: 0.46–40.01). For ELISA seropositivity, the odds ratio was 2.69 (95% CI: 0.49–14.64). While seropositivity was observed to be numerically higher in the CKD group, neither the comparison based on ELISA (*p* = 0.434) nor the comparison confirmed by Western blot analysis achieved statistical significance. The wide confidence intervals, which included the null value (OR = 1), indicate considerable statistical uncertainty due to the limited sample size (Table 3).

All four Western blot-confirmed seropositive CKD patients lived in rural areas distant from urban centers. These individuals were engaged in farming and/or animal husbandry and reported a history of repeated tick exposure. Furthermore, none of the participants with confirmed seropositivity through Western blot assays reported a prior diagnosis of Lyme borreliosis, exhibited symptoms indicative of previous Lyme disease, or received any treatment specifically targeting Lyme disease. The single Western blot-positive control participant resided in an urban area but reported regular weekend visits to a village with hazelnut orchards. This individual did not engage in animal husbandry and did not report a history of tick exposure.

## 4. Discussion

Environmental and climatic changes may influence tick distribution, abundance, and seasonal activity, potentially contributing to changes in Lymeborreliosis risk and recognition [2,20,21]. If not treated on time, Lyme disease can have significant pathological effects on multiple organ systems, including the skin; musculoskeletal system, especially in the joints; cardiovascular system, mostly the heart; and central nervous system, potentially leading to a multisystemic clinical presentation [22,23].

The existing literature on renal involvement in Lyme disease among humans is notably limited, consisting primarily of individual case reports and small case series. Documented clinical manifestations associated with Lymeborreliosis include membranous glomerulonephritis, membranoproliferative glomerulonephritis, IgA nephropathy, and minimal change disease. The precise prevalence of glomerulonephritis and other renal manifestations associated with Lyme disease has not been adequately characterized, and these figures may be significantly underestimated. This limited body of work highlights the need for further investigation to better understand the implications of Lyme disease for renal function and the underlying pathophysiological mechanisms [24]. In this context, our findings contribute to the limited body of evidence investigating the potential correlation between *Borrelia* exposure and chronic kidney disease.

Renal involvement, specifically Lyme nephritis, is well-documented in canine populations; however, the existing literature concerning human cases remains notably scarce, and the cause is still unclear. This paucity of data underscores the necessity for further research to elucidate the mechanisms, prevalence, and clinical implications of Lyme nephritis in humans. The precise prevalence of glomerulonephritis and other renal manifestations associated with Lyme disease in humans remains in adequately characterized. This underestimation is likely attributable to the intricate, chronic, and recurrent nature of the clinical presentation, which may result in diminished clinical suspicion among healthcare practitioners. Consequently, these renal complications may often go unrecognized and underdiagnosed within the broader context of Lyme disease. To date, there is a notable absence of comprehensive studies examining the relationship between Lyme disease and chronic renal failure in humans. The present investigation seeks to serve as a pioneering effort in this under-researched area, contributing valuable insights and fostering a deeper understanding of potential correlations between these two medical conditions.

In the comprehensive assessment conducted among both patients and control subjects, the prevalence of Western blot-confirmed anti-*Borrelia* IgG positivity was found to be 5.55%. Out of a total of 90 participants, the ELISA identified seven individuals as positive, representing 7.78% of the sample population. Additionally, Western blot testing confirmed five individuals as positive, which constitutes 5.55% of the participants. Başbulut et al. conducted a seroprevalence study within the same geographic region, reporting a seropositivity rate for anti-*Borrelia* IgG of 4.1% as determined by ELISA and 3.3% through Western blot analysis (25). Our investigation revealed elevated rates, which we ascribe to increased seropositivity observed within the cohort of individuals with chronic renal failure. In our study, we observed that the seroprevalence of anti-*Borrelia* IgG antibodies among patients with chronic renal failure was measured at 11.1% using the ELISA method and 8.9% via Western blot analysis. In contrast, control subjects exhibited lower seropositivity rates of 4.4% and 2.2%, respectively. Although seropositivity was numerically higher in the CKD group (8.9%) than in the control group (2.2%), the difference was not statistically significant. Given the small number of seropositive individuals, this observation should be interpreted as exploratory and warrants confirmation in larger studies.

Drake et al. [17] demonstrated that dogs seropositive for *Borrelia burgdorferi* in endemic regions face a heightened risk of developing chronic kidney disease. This finding underscores the importance of diligent monitoring of renal health in affected canine populations. It highlights the necessity for further investigative studies to explore the potential association between *B. burgdorferi* seropositivity and renal pathophysiology. This observation is consistent with our research findings, indicating that a more in-depth exploration of the association between *B. burgdorferi* seropositivity and chronic kidney disease in humans is warranted. Further investigation into this relationship could reveal significant insights into the potential implications of Lyme disease on renal health.

Lyme borreliosis exhibits no significant variations based on demographic factors, including age, sex, or race. Risk factors associated with infection by *Borrelia burgdorferi* encompass various occupational and recreational activities. These include involvement in farming, animal husbandry, dairy production, and slaughterhouse operations, as well as direct exposure to ticks. Such activities significantly heighten the likelihood of encountering the vectors responsible for Lyme disease transmission. Increased seropositivity has been observed among individuals residing in rural environments and elevated terrains (≥400 m above sea level) [25,26]. Our investigation revealed that the seropositivity rates were somewhat elevated. The higher rate observed in the present cohort may reflect the inclusion of patients with greater rural and occupational exposure; however, the small sample size and the absence of systematic exposure assessment for all participants preclude firm conclusions regarding the reasons for this difference.

Due to logistical constraints, a comprehensive individual screening of all study participants for risk factors associated with *B. burgdorferi* infection was not feasible. Nevertheless, within the cohort of patients diagnosed with chronic renal failure who exhibited seropositivity for anti-*Borrelia* IgG, an assessment of risk factors was conducted. It was determined that all individuals in this subset were actively involved in agricultural practices and animal husbandry, residing in a rural environment. Additionally, these patients presented with a documented history of frequent exposure to ticks, further underscoring their elevated risk for *B. burgdorferi* infection. Therefore, the observed seropositivity in this subgroup may primarily reflect environmental and occupational exposure rather than CKD-related biological factors. This possibility should be considered when interpreting the findings of the present study.In endemic or high-exposure settings, clinicians may consider Lyme borreliosis in the differential diagnosis of selected patients with unexplained renal findings, particularly when a compatible exposure history or systemic manifestations are present. Future longitudinal studies are needed to determine whether *Borrelia* exposure has any role in the development or progression of chronic kidney disease and to evaluate the potential impact of appropriate treatment strategies. This evaluation could provide critical insights into prevention strategies and the long-term outcomes associated with various interventions.

Although Lyme nephritis is a well-established condition in canine populations, there remains a paucity of literature regarding its occurrence in humans. This highlights the need for further investigation into the pathophysiology, clinical presentation, and potential treatment strategies for Lyme nephritis in humans.

The study has several limitations. The sample size was small, and the study may have been underpowered to detect statistically significant differences. In addition, the relatively small sample size precluded multivariable analyses to adjust for potential confounding factors. A further limitation of this study is that potential risk factors for *Borrelia* exposure, including rural residence, occupational exposure, animal contact, and history of tick bites, were not systematically collected from all participants. Therefore, their potential contribution to seropositivity could not be evaluated. In addition, the relatively small sample size precluded multivariable analyses to adjust for potential confounding factors. Furthermore, the relatively short study period and cross-sectional design preclude evaluation of long-term outcomes and of temporal relationships between *Borrelia* exposure and chronic kidney disease. Therefore, no conclusions can be drawn regarding causality, disease progression, or the potential long-term clinical significance of the observed serological findings. Despite this limitation, the study addresses an underexplored question and may serve as a basis for future research. Larger multicenter studies with matched controls, systematic risk factor assessment, longitudinal follow-up, and detailed renal phenotyping are needed to determine whether *Borrelia* exposure is associated with unexplained CKD or whether the observed difference primarily reflects environmental exposure patterns in rural populations. The Western blot-positive control participant also reported regular visits to a rural area, suggesting that environmental exposure may contribute to anti-*Borrelia* seropositivity irrespective of CKD status.

Additionally, although none of the participants with Western blot-confirmed seropositivity reported a previous diagnosis of Lymeborreliosis or treatment specifically directed against Lyme disease, detailed information regarding potential prior Lyme disease–related symptoms, treatment regimens, and treatment duration was not systematically collected using a standardized questionnaire. This limitation restricts a more comprehensive assessment of the clinical relevance of the serological findings.

## 5. Conclusions

Anti-*Borrelia* IgG seropositivity was numerically higher among patients with CKD of unknown etiology than among healthy controls, although the difference was not statistically significant. Among the four Western blot-positive CKD patients, all lived in rural areas distant from urban centers and reported farming and/or animal husbandry activities with repeated tick exposure. These characteristics were common to all seropositive CKD patients in our cohort.

These findings should be considered preliminary and hypothesis-generating. Further large-scale, risk-factor-adjusted, and longitudinal studies are needed to clarify whether prior *Borrelia* exposure is independently associated with unexplained CKD or its progression in humans.

## Figures and Tables

**Table 1:** Etiological distribution of hemodialysis patients screened during the study period.

Etiology	Number	Percentage
Diabetes mellitus	54	33.5%
Hypertension	48	29.8%
Glomerulonephritis/pyelonephritis	2	1.25%
Obstructive uropathy	2	1.25%
Amyloidosis secondary to familial Mediterranean fever	1	0.63%
Vasculitis	1	0.63%
Renal artery stenosis	1	0.63%
Indeterminate etiology	52	32.3%
Total	161	

**Table 2:** Baseline characteristics of the study groups.

Characteristic	CKD Group (n = 45)	Control Group (n = 45)	*p*-Value
Age, years, mean ± SD	60.07 ± 11.74	57.42 ± 7.79	0.212
Female, n (%)	18 (40.0%)	19 (42.2%)	0.830
Male, n (%)	27 (60.0%)	26 (57.8%)	

SD: standard deviation; CKD: chronic kidney disease. Age was compared using the independent samples *t*-test; sex distribution was compared using Pearson’s chi-square or Fisher’s exact test, as appropriate.

**Table 3:** Comparison of Anti-*Borrelia* IgG seropositivity between groups.

Variable	CKD Group (n = 45)	Control Group (n = 45)	OR (95% CI)	*p*-Value
ELISA seropositivity	5 (11.1%)	2 (4.4%)	2.69 (0.49–14.64)	0.434
Western blot-confirmed seropositivity	4 (8.9%)	1 (2.2%)	4.29 (0.46–40.01)	0.361

Abbreviations: ELISA, enzyme-linked immunosorbent assay; WB, Western blot; CKD, chronic kidney disease; OR, odds ratio; CI, confidence interval. Odds ratios compare the odds of seropositivity in the CKD group relative to the control group. Positive ELISA results were evaluated by Western blot confirmation. Between-group comparisons were performed using Pearson’s chi-square or Fisher’s exact test, as appropriate.

## Data Availability

The data supporting the findings of this study are available from the corresponding author upon reasonable request.
